# Rapid qualitative health research from the Global South: Reflections and learnings from Argentina, Brazil, Chile, and Mexico during the COVID-19 pandemic

**DOI:** 10.3389/fsoc.2022.983303

**Published:** 2022-09-28

**Authors:** Itzel Eguiluz, Anahi Sy, Eugenia Brage, Marcela González-Agüero

**Affiliations:** ^1^Instituto de Investigaciones Económicas, Universidad Nacional Autónoma de México (UNAM), Mexico, Mexico; ^2^Departamento de Salud Comunitaria, Instituto de Justicia y Derechos Humanos, CONICET, Universidad Nacional de Lanús, Buenos Aires, Argentina; ^3^Centro de Estudos da Metrópole (CEM, Cepid, FAPESP), Universidade de São Paulo, Centro Brasileiro de Análise e Planejamento, São Paulo, Brazil [Processo Fapesp n° 2019/13439-7]; ^4^Escuela de Enfermería, Pontificia Universidad Católica de Chile, Centro Colaborador OPS/OMS, Santiago de Chile, Chile

**Keywords:** COVID-19, health emergencies, Latin America, qualitative research, rapid qualitative research, Global South, reflexivity, positionality

## Abstract

The objective of this paper is to provide insights into our experiences undertaking qualitative rapid research in Latin American contexts based on fieldwork from Argentina, Brazil, Chile, and Mexico. We focus on the insights and learning processes that emerged from our research teamwork during the COVID-19 pandemic. Our research projects are part of an international collaboration led by the Rapid Research Evaluation and Appraisal Lab (RREAL) to explore the experiences of COVID-19 Frontline Healthcare Workers. The analyzed experiences not only rely on the local studies but also on our reflections as a group of Latin American researchers collaborating along with an international team. Qualitative research has an important and long-lasting tradition in Latin America. However, healthcare professionals are still reluctant to use these methods. We highlight tensions and dilemmas that have emerged from our own empirical experience: First, the time for research ethics committees to evaluate the protocols; second, the difficulties in accessing funding to undertake research due to the lack of financial opportunities; third, having to decide the language of our publications. That is just the tip of the iceberg that allows us to show inequalities in the conditions under which scientific knowledge is produced between the North and the Global South. Following these points, our text explores the tension between the urgency to conduct rapid research and the multiple difficulties when undertaking it during the pandemic. It is important to point out that the problems we faced already existed before the sanitary emergency, being magnified by the former. At last, our conclusions delve into the reflexive process we, as a team of female researchers, undertook to explore the differences and similarities of our experiences. This analysis allowed us to solve obstacles and dilemmas when doing research. The winding road we describe here serves as an example for other research teams when planning and undertaking rapid qualitative research during future pandemics.

## Introduction

This paper's main objective is to provide insights from our experiences undertaking rapid qualitative research (RQR) in Latin American countries such as Argentina, Brazil, Chile, and Mexico. As Latin American female researchers we reflect on the tensions and learning processes that emerged from our teamwork during the COVID-19 pandemic. The research projects on which we based our discussion are part of an international collaboration led by the Rapid Research Evaluation and Appraisal Lab (RREAL, UCL), focused on exploring the experiences of frontline healthcare workers during the pandemic in different countries, reaching up to 22 teams worldwide.

Within the framework of this international collaborative project, which pretended to draw a global picture of the working conditions of healthcare workers, arose the need to articulate, compare and analyze experiences between countries in the region. This was needed even though there were important differences in the way each country handled the pandemic and in the articulation of cooperative health strategies (Basile, 2020).

As Vindrola Padros and Johnson have shown, the 22 countries involved in this network experienced different situations during the research, each team was “shaped by delays generated by ethics review committees, restrictions that prevented access to medical facilities and staff, limited budgets for research and the pressures researchers were facing in their own lives (uncertainty, fears, childcare issues, illness, and bereavement)” (Vindrola-Padros and Johnson, 2022, p. 3). Taking this into consideration, in this paper we analyze the experiences of researching the Global South.

Thanks to these dialogues, common aspects emerged around the ways of doing research. This collaborative research experience has become an opportunity to question and reflect upon the task of undertaking research from the “Global South,” given the contrast with other experiences from teams based in central countries. These questions and reflections have led us to look at our experiences collectively. In this article, we analyze some of these points.

Global health is a relatively new approach in the field of public health. This perspective, which supposedly defines a global agenda based on the health needs of the population of the entire planet, above the interests of particular nations, however, has received deep criticism from countries of the Global South, arguing that its scope does not it is only limited and linked to specific and decontextualized interests, but also reproduces the perspective of the Global North and not a perspective of rights, justice, equity and global political determinants of health.

Global health promoted through international organizations and based on multilateral and unilateral cooperation agreements that operate vertically on the territories called the “Global South,” globally defines its objectives, which are not necessarily suitable for the countries of the global south, reproducing, from this colonialist logic (Fleury, 2001; Rovere, 2014). It is worth adding that the literature on the subject produced in the last two decades comes, fundamentally, from institutions based in central countries, with the English language being predominant. It is from this group of countries and their academic institutions that the very notion of global health was installed and consolidated to solve health problems at the international level.

Advocating for the health sovereignty of our territories, and from an epistemological position from the south (de Sousa Santos, 2009, p. 368), some approaches propose a decolonial and epistemological turn in terms of a New South-South International Health (Basile, 2018). This last author warns that the discussions on the intellectual and political construction of international health must carry out two simultaneous intellectual exercises: the internal criticism of the hegemonic logics imposed from the Global North, and the formulation of interests and strategies of the South based on the autonomy, geopolitics, emancipation, history, and culture (p. 8).

Taking this into consideration from an epistemological position from the South, we analyze the ways of making and producing knowledge, highlighting not only the differential conditions in which we carry out our work, about the countries of the Global North, but the theoretical, methodological, and political potential that derived/emerged from our “subaltern” condition, which reflects a political imagination beyond the solutions and alternatives thought from and for the north and applied to our territories.

In this way, we aim to unveil some of the North-South inequalities and the different ways of doing research regarding not only theoretical backgrounds but tools and opportunities from our contexts, highlighting, as well, some tensions and dilemmas that have emerged from our own empirical experience undertaking RQR during the pandemic.

Qualitative research on health issues has an essential and long-lasting tradition in Latin America. However, some scientists, and experts in clinical, biomedical, and even social sciences are still reluctant to use these kinds of methods, even when there is a local social-epidemiology tradition developed very closely to social sciences methods and techniques. Although its importance in health research is recognized, due to its contributions to generating concrete and useful insights in less time during a crisis, there is still some mistrust and resistance to its application, especially concerning the quality of these investigations (Vindrola-Padros et al., 2021). In Latin American Countries there is no large trajectory doing RQR. That is mainly linked to Applied Anthropology which has a limited expression reduced to some teams' trajectories (see Freidenberg, 2008). This type of approach is usually rejected in the fields of research in the social sciences, especially anthropology. There is frequently an automatic association of rapid research as a “quick and dirty” exercise, as has been described in other countries (Vindrola-Padros and Vindrola-Padros, 2018; Vindrola-Padros, 2020).

A systematic review on the subject (Vindrola-Padros et al., 2021) shows that there are still questions related to the use of these methods, especially concerning their suitability and the reliability of the data, and the degree of use of the results obtained through their use. It is not the objective of this article to go into depth about these postulates. Rather, what we are interested in pointing out is how the question of “fast” enters into tension with logic, times, and concrete possibilities of doing research in our contexts and shows that, despite the limitations, the quality of research has not been diminished. In this sense, the rapid adaptability of research teams, the rigorous analysis, and the high level of production, even with few resources, stand out.

It is increasingly identifying themes that encourage dialogue with counterparts in the Global North, suggesting ways to use the knowledge that could link anthropology from the North and the South in the American continent (Freidenberg, 2022). Anyway, the implementation of qualitative research in the context of Latin American countries, as has been described for other countries, continues to lag in the delivery, credibility, and timeliness of findings when compared with other research designs (Vindrola-Padros et al., 2020).

Concerning this, respect for Rapid Qualitative Research (RQR) and the evaluation approaches, a systematic review on the use of Rapid evaluation in health care (Vindrola-Padros et al., 2021) showed that the most frequent reason for the use of these approaches was the need to report results of the findings to inform decision making, established programs or the provision of services. When discussing Rapid Qualitative Research, it is relevant to remember Beebe (2014) who argues that it is not rushed research, it is rapid research. Although, it is not the same doing RQR in our countries as in central ones, for example, time in producing and publishing articles differs and also does the approach to the field. Some of the dilemmas we discuss are closely related to the fact that strategies guided by the central countries colored the responses developed by the different countries to the pandemic. Advocating for health “from the south,” Basile (2020) points to an issue that also crosses our position as researchers from countries of the Global South. It is necessary to take into consideration the impacts of the geopolitics of power and health knowledge in coping with the global SARS-CoV-2 pandemic.

## Doing rapid qualitative research in Latin America

### Rapid qualitative research: Waiting for the Ethics Committee resolution

The decision to undertake a research project of any kind and at any time requires an ethics committee's favorable resolution. In our countries, most committees are used to evaluate laboratory-based and clinical research mainly, so, qualitative approaches sometimes generate questions and doubts that contribute to the slowing down of the evaluation process. In ethics committees, there is a predominance of researchers who come from the natural sciences and biomedicine, which biases the evaluations in some situations, for example, asking qualitative research for elements that are not it is own (even epistemically), demanding clarifications that are taken for granted in natural sciences or predicting situations of risk that are exaggerated and contrary to what happened in the investigation, as can be seen in the following case.

Brazil has a unified ethics committee system, which is called “*Plataforma Brazil*.” All projects must be uploaded to the website and once this first step is completed, the project will go through a series of steps until the documentation is ready to proceed to the evaluation phase. After this instance, the project is sent to an Ethics Committee, depending on the region where the project will be developed. For this first stage of the process, it is necessary to have all the prior authorizations from the health centers where the research is proposed to be carried out.

While there was also a resolution in Brazil that streamlined COVID-19 projects, we experienced a particular delay in getting approval, which took more than 4 months from the moment in which the application was lodged. The reasons for this delay were the successive requests for explanations regarding “how we would act in the face of possible sensitivities of health professionals,” which had already been previously addressed, following relatively standardized guidelines of qualitative research ethics. However, due to the disagreement of the evaluators regarding this point, we proposed offering psychological aid to participants through a program designed by the health system to provide psychological and psychiatric support to health professionals who work in the public system. This was also not accepted by the committee. Finally, we had to incorporate a psychologist into the team, who assumed the role of providing psychological assistance in the face of any eventuality. It is worth mentioning that the investigation proceeded without incidents. All the people who chose to participate in the study authorized the recording of the interviews, except one person. Contrary to what was argued by the ethics committee, there were no situations of discomfort on the part of the participants, quite the contrary. They appreciated having the opportunity to be heard and narrate their experiences in the pandemic.

In this context, emerges the question: To what extent certain ethical safeguards were insisted on when it comes to qualitative research that is not considered in quantitative research? And in that sense, comparing committee evaluations in different countries we saw different criteria and feedback questions around projects with the same methodology.

In some countries like Argentina, the Ministry of Health passed a resolution during the pandemic to fasten the ethics evaluation process. There were no extra requirements to get the approval. This allowed researchers to defer the signing of the informed consent form when their projects did not involve interventions on a person's body, which is the case of qualitative research^1^ This contributed to speeding up the process of gaining ethics approval for RQR.

The Chilean case was like the Argentinian case, as Ethics Committees also began to function with a fast-track process for studies focused on tackling different dimensions of the pandemic. This strategy was put into place mainly to facilitate the timely implementation of clinical studies that aimed at trialing new drugs and therapies to address the population's health needs. The fast-track revision process did not involve a less thorough evaluation, but it helped Ethics Committees to prioritize those applications that had a clear aim of addressing the challenges posed by the pandemic, over those studies that were concerned with other topics.

The qualitative study conducted by the Chilean team benefited from this process as its main objective was to explore healthcare workers' and patients' perceptions of COVID-19 and the health response in Chile during 2020–2021. The research team collected data in five regions of Chile, which at the time had the highest number of cases of COVID-19. From now onwards, we will refer to this study with the name “ExpCOVID.” Considering the sanitary restrictions imposed by the pandemic, the team decided to undertake all interviews with frontline healthcare professionals via telephone. Additionally, the consent form was designed on a website, allowing potential participants to review the characteristics of the study and accept participating in it online. Only after accepting taking part in the study, did the research team receive a notification with the participant's contact details.

After gaining consent and throughout the implementation of the ExpCOVID project, the team applied for amendments on three occasions. These amendments allowed the team to adjust the study according to the dynamic context generated by the pandemic, but also to devise new strategies to improve the visualization of the study and thus, the recruitment of new participants. All amendments were approved within the following 2 weeks after being lodged, as Ethics Committees hold weekly meetings during the pandemic.

The first amendment focused on improvements in the interview guide and the website. The second one responded to epidemiological changes and sought to increase the number of regions where data was collected. It also incorporated the option to undertake interviews online using the Zoom platform. The final amendment focused on new strategies for recruitment. This was one of the main challenges faced by the team, as frontline workers were already extremely tired due to their workload, the nature of the disease, and the ever-changing contexts (we have unpacked this in another publication, see Brage et al., 2022). Thus, inviting them to take part in an interview was, in a sense, extra work for them, but we believe that it also represented an opportunity to reflect on their everyday routine at work and home, and unpack a deeply emotionally and physically charged experience. At the end of many interviews, participants thanked the interviewers for listening, stating that the interview had been a positive experience for them.

In other cases, like the one from the Mexican team, there is no national regulation for research ethics committees, for non-clinical research, it depends on each institution, public or private. This lack of consensus leads to two main problems: in the first place a lot of health-related institutions do not evaluate non-clinical research in their ethics committees and second, there is almost no place for independent research to get an ethical committee evaluation, therefore sometimes it is not possible to collaborate in international research projects. These issues lead us, as a Mexican research team, to work with no official ethics approval. To comply with international standards, we delivered information letters to all our participants, explained, and used an informed consent form, and kept interview transcripts utterly anonymous from the moment we started fieldwork through the publication. Nonetheless, this lack of ethical accountability could lead to the impossibility of applying for funds and participating in international research teams in the future.

The political moment that each of the countries experienced configured different ways of dealing with the pandemic, generating, and exacerbating uncertainties (Brage et al., 2022). In that regard, we can ask ourselves about the best alternatives to guarantee the conduct of ethical research globally, considering so many different experiences and contexts. In contrast, the ethical behavior of the researchers should follow similar principles (considering cultural adaptations according to the context). Even though there are different experiences in different ethical committees, for example, based on the way they are configured, oriented to biomedical or laboratory-based research, or oriented to include social sciences. Based on our mixed experiences, probably it is necessary to open a discussion about the tensions that emerge from doing qualitative research in such circumstances, which is different from biomedical research, and thus to create more pertinent and clear criteria that may allow teams from different disciplines to carry out their studies without this kind of barriers.

### Local experiences of seeking funding

In “normal” circumstances, that is, in non-pandemic contexts, accessing funding for undertaking research projects takes time, persistence, and great effort. Regularly, researchers may need between 6 months and a year to apply and receive funds for their proposals if their application is successful. In pandemic circumstances, these timelines were untenable, as the context required faster processes to apply and receive funding. The health emergency became a source of extra funds and accelerated mechanisms of research project evaluation.

At the beginning of the pandemic, this phenomenon was mainly seen from an epidemiological viewpoint, therefore most of the funds were intended for epidemiological and medical-clinical research, although social scientists were participating in the analysis of the sanitary crisis, contributing first with speculative knowledge, which was necessary under those circumstances but not oriented to solve empirical problems. Later, when the profound effects of the pandemic on social life were highlighted, and the syndemic character of the crisis was acknowledged, the necessity to involve the social sciences in the analysis became urgent and desired. These not only contributed to a better understanding of the social inequalities that appeared during the pandemic, but also unveiled people's living conditions, experiences, and perspectives, especially in low-income countries (like Latin America ones). As Pickersgill et al. (2022, p. 1) have stated: “social scientific research on COVID-19 has increased as the pandemic has evolved.”

This broader perspective enables the recognition that social sciences research is essential in the context of pandemics. Some countries organized special calls for social sciences research grants. In Argentina, for example, after 6 months or more of only financing clinical or epidemiological studies, there was a new interest in social sciences, seeking for knowledge-oriented proposals to comprehend people's behaviors, the impact of pandemics in poorer families, people's strategies to solve daily problems in this context, their demands to the state, the issue of food security and violence associated to the pandemic, among others. Suddenly it became urgent “to know more” about people's everyday experiences, including those of healthcare workers, which constituted a change in the previous focus on the virus behavior. As a result of that interest, the Argentinian team received a grant from the National Agency of Research, Development, and Innovation (PISAC-COVID-19Agencia I+D+i announcement, 2020)^2^ We would like to highlight that this research fund particularly targeted women in sciences, promoting gender equity in research. While the grant offered financial support to undertake part of the study the Argentine team received the funds in March 2021, after being working on the topic since March 2020 (as often in social sciences there are teams with more people working than getting paid for).

Regarding the experience of the Mexican team, two independent research groups participated in the collaboration with RREAL. The first team worked at the beginning of the pandemic to develop a public policy analysis to identify health inequalities between public and private institutions, exemplified by their response during the COVID-19 epidemic in Mexico. This team was led by researchers from an NGO^3^. Nonetheless, the other team which developed two projects one with frontline healthcare workers and one with emerging adults was not funded, and the team worked on these projects due to personal interest and *ad honorem*. At some point, the project received the symbolic support of the Public Health Mexican Society, as this organization sponsored the study by lending its name to accomplish the “professional adscription” of the project, increasing its credibility. This was crucial to undertake the recruitment process, as the team could show in ads shared on their social networks that this organization sponsored the study, becoming more attractive to recruit participants.

In the case of Brazil, the project was developed based on previous agendas of researchers from the institute (*Centro de Estudos da Metrópole*). In this way, the team did not aspire to gain specific financing to undertake the study, but instead, they proposed to complement each other and broaden their approach to contemplate the objectives of the collaborative project. In the practice, this meant that the researchers who were already collecting data for other studies accommodated their fieldwork to include the objectives sought by RREAL. This also meant submitting ethical amendments to already approved studies.

The Chilean study began as all the other Latin American projects described above: lacking funding. However, during the early stages of this project, we benefited from funding from the Chilean National Agency of Research and Development (*ANID* in Spanish). In late April 2020, *ANID* launched a funding scheme “for the rapid allocation of funds for research projects on Coronavirus (COVID-19).” The purpose of this scheme was to finance initiatives linked to the diagnosis, control, prevention, treatment, monitoring, or any other aspect related to the pandemic and its consequences, from a scientific, technological, sanitary, social, economic, cultural, and humanistic perspective^4^. In the 23 days in which this call was opened, *ANID* received more than 1,000 proposals and only 63 of those received funding, being ExpCOVID one of those^5^ We believe that our project was competitive because when we applied for funding, we already had ethics approval to undertake the study and a strong international collaboration with RREAL, which was a requirement of this funding scheme. It is important to consider that the small number of grants allocated demonstrated, on the one hand, the great interest of local research teams to undertake projects connected to the pandemic and their ability to prepare a proposal with very short notice. On the other hand, the 63 grants allocated highlighted the small chance most researchers have when applying for funding.

We cannot ignore the fact that aspiring for funds always takes considerable time and dedication that, in the context of the pandemic, overlaps with the infinity of tasks that all of us carry out, as well as with the tasks of reproduction necessary for sustaining life. In this way, we did not spare extra time to raise funds for these projects and we decided to juggle the talents, knowledge, and abilities each member of the team had, optimizing time, energy, and resources, something that women and dissidents know how to do quite well in our daily lives.

### Local Latin American teams meeting global ones

The pandemic brought to the fore the concept of Global Health, as its impact and long-term consequences went beyond geopolitical boundaries. Very quickly, on the 30th of January 2020, the International Health Regulations Emergency Committee of the World Health Organization declared COVID-19 a Public Health Emergency of International Concern (PHEIC). This status is used for under exceptional circumstances where there is a clear “public health risk to other States through the international spread of disease” and which require a “coordinated international response” (Wilder-Smith and Osman, 2020, p. 1). As a field of research and practice, “Global health emphasizes transnational health issues, determinants, and solutions; involves many disciplines within and beyond the health sciences and promotes interdisciplinary collaboration; and is a synthesis of population-based prevention with individual-level clinical care” (Koplan et al., 2009, p. 1995). Thus, the research projects our teams undertook contribute to this field and at the same time, are marked by its characteristics and emerging tensions.

In recent years different concerns regarding the asymmetries that emerge from Global Health have arisen (Montenegro et al., 2020), which are relevant to our argument, in the sense that, on the one hand, the pandemic uncovered how the global North and the South communicate with each other, which voices are considered *valid* and how recommendations (for research and practice) designed in the North not necessarily apply to the South. On the other hand, the pandemic showed patterns and strategies for establishing research-related relationships between academics from the North and South.

As stated by Seye Abimbola, “there is a problem of gaze at the heart of academic global health” (2019, p. 1), referring to the issue of identity and positionality. Knowledge production is interwoven with who we are as researchers, from *where* we write (in an epistemological and geographical sense), and to whom we write. Thus, it is relevant to explore the academic relationships that emerge from Global Health-related topics such as the pandemic, grappling with tensions that may appear from the management of projects, timelines, language in which we write, rules, frameworks, and available resources, —or their lack thereof.

The research initiative that brought the authors of this paper together emerged during the pandemic from a social sciences research team based in the UK that invited researchers from around the globe to participate in an international research network interested in the experiences of healthcare workers during the COVID-19 pandemic. This network is coordinated by RREAL, at the University College London. Thus, our involvement in this network took place through a North-South invitation to collaborate and share our findings and experiences. Considering that the pandemic first hit the North, it was expected that academics based there began designing and implementing research projects to explore the complexity of the pandemic before those located in the South. But we also believe that globally, the resources (financial, human, technological, and social) available for researchers and academics are unequally distributed. In this respect, it is interesting to mention that the network does not offer any kind of financing for the projects, and some of the national teams that participated neither have access to funding in their countries or to human resources to undertake the research projects, therefore an important part of the work undertaken by our teams was “volunteer work” for the whole project or at least for most of it varying from team to team.

That is representative of inequalities in knowledge production conditions. While in the North the teams are financed and very well-constituted from the first day of the research (or almost that seems so), in the South the conditions are very different, the teams are hardly financed and many times we finance our research from our salaries. That has an impact on the possibility of fast data collection, analysis, discussion, and dissemination. In this way, we ask ourselves whether rapid research appraisal is compatible or not with the South constitution of research teams and the working conditions we have.

Take as an example the situation of the Chilean team, which had 12 members, all of their academics with different degrees of experience in undertaking qualitative research. Of the team, only the principal researcher (the only male in the team) and the main co-researcher had enough allocated time during working hours to conduct this study (5 and 4 h, respectively), while all the other researchers only had 2 h weekly dedicated to the project. Often, this time was insufficient, which pushed researchers to use their time to undertake research activities. However, their enthusiasm and commitment to the topic were outstanding, becoming a facilitator to complete the project according to its timeframe. One aspect that helped the team to function effectively was the participation of three postgraduate students (two males and a female student) who completed their master's thesis as part of this project.

In Argentina, in 2020 the team was the same that was doing research at hospitals previously, a team of four women, including the main researcher, the only one with relative stability in her job as a researcher. Two others were postgraduate students doing their master's thesis and another one depended on funds from local projects. By November 2020, the latter decided to leave the study to take care of her children and work freelance from home. At that time, another postgraduate student was incorporated into the team to do her master's thesis related to this project. Also, a postdoctoral student was incorporated due to his interest in collaborating, despite the fact of erratic financial resources. In this uncertain and precarious context, it was difficult to enlarge and hold on to the research team.

In the RQR conducted by the Mexican team, there were three researchers, all of them women, who had different roles: one research assistant who was also a clinical physiotherapist, one who worked as a project manager while undertaking a Master's program, and an independent researcher who held a Ph.D. Only the principal researcher could allocate more than 3 daily hours to the project, while the other two researchers worked on it mainly during their time. Due to the nature of the study, most of the interviews were done at the best time possible for the healthcare workers, which meant the research team had to work during weekends and at night, and as said before totally *ad honorem* as we were convinced of the importance of the project.

The Brazilian team was made up of three main researchers, all of them women who, as mentioned above, joined their health research agendas to carry out the project, each of them with funding from other research under development. It is worth clarifying that the material collected through face-to-face interviews was possible thanks to the fact that one of them was doing fieldwork in a healthcare center as part of her postdoctoral research. In other words, the interviews could not have been carried out if it had not been for the financing of the postdoctoral fellowship. Likewise, it is worth mentioning that in some stages two of the researchers diverted resources from their research projects to finance, for example, a master's student who revised public policies to the pandemic.

### Publishing debates: Cost and language

Most of the academic journals that are well-indexed and positioned on rankings, for example, h-factor, mainly publish in English, therefore, we Latin American researchers are academically better evaluated when publishing in these journals, even when in some cases our results are more pertinent, useful and suitable for a Spanish-speaking audience, where we can reach a broader audience, but probably with fewer citations (Franco-López et al., 2016) that are also evaluated in some contexts.

As researchers we need to decide in which language we want to publish, in our case Spanish, Portuguese or English, mainly. If we decide to do it in English, then we must consider if we can write the text directly in this language, which implies evaluating our ability to write in another language and the extra time we need to do that. If we decide to write it in English, we may need an expert to revise the text, and if we do it in Spanish we will need funding for translation, as we said before, this may not be a possibility due to the lack of funding. Another issue surges when we translate or adapt interview guides, scales, other research tools, and, regarding qualitative research, the translation of participants' quotes, which may contain slang. With translation comes the risk of losing meaning or usefulness.

Publication in these international journals comes with other problems, sometimes the publication costs charged by some English-language journals are higher than Spanish-language ones, and in most cases, the latter does not have a publication fee. The costs for some international English-language indexed journals usually are charged in US dollars or UK pounds, the conversion rate results in very high publication costs that may be equal to a researcher's monthly salary, one study shows that an average cost is about US$400 (Grossmann and Brembs, 2021). Even when the journal does not have a publication fee, it may have the option for open access or fast-track reviews, with added cost. Paying or not for open access also has consequences for readers and researchers, the former may have to pay high fees to access the articles as their universities may not have access to the journals or their more recent editions, and the latter may be affected because their research may lose diffusion and therefore may have fewer citations.

Although we know that publications in English are better evaluated in the academic career, a discomfort crosses us all equally, concerning this point. It is not something new and we know that although we are required to write in English our research is more valuable in our local languages. This is not something that we as researchers ignore, on the contrary, we work double, we must do the translation exercise, try to express ourselves in correct, academic, and professional English our ideas, striving to transmit practices and meanings from our “peripheral” environments so that they are read in the hegemonic language. At the same time, we must rewrite to fulfill our commitment: create and publish local knowledge.

Another difficulty present when trying to publish our work, especially when doing RQR from our experiences is time. We are saying that this kind of research is Rapid, remember, not rushed but rapid, and even with the team members' number, funding, and other restrictions when conducting the studies, we were doing RQR until the publication part arrived. Publishing in a free-cost journal in Spanish could lead to long waiting times, this, of course, depends on the journal and other issues, nonetheless in our experiences from this specific project their articles have been almost a year in the process, and some already accepted for publication, while there are multiple publications from the same project in other languages already published, some of them months ago.

Regarding publications, the Brazilian team faced a triple effort by having to deal with three different languages. In the first place, the joint publications were in English, as were the materials provided for the development of the research and the preparation of material to be presented at the meetings. On the other hand, in collaboration with other Latin American countries, this material collected in Portuguese had to be translated into Spanish, in the same way as if joint publications were intended. Finally, regarding the ethical and scientific commitment to return results and spread knowledge, these should be published in Portuguese and preferably in Brazilian journals. In short, the Brazilian team faces multilingual challenges when it comes to publishing and, not having the funding for it, which leads to delays in publishing.

Finally, we want to leave open the ethics discussions about when to publish in English, as we are doing in this article. Is it ethical to discuss power, decolonization, and other issues in our countries and the global sphere usually from English? Is it contradictory? Maybe one solution could be the controversial double publishing, journals could become multilingual, journals could offer financial aid or free open distribution when the author contributes with some peer review or could offer translation-language reviews.

## Reflexive process as a team of women who are also researchers

The reflexive process as a team of women exploring the differences and similarities of our experiences from different countries converges on care, which challenges us personally as women during the pandemic while it favors empathy with the people interviewed -women, mothers, caregivers, workers, in some cases household breadwinners. This task that finds us as a female gender with the interviewees leads us to problematize the working conditions and the conditions of knowledge production (and at the same time, care).

The pandemic has exacerbated pre-existing structural conditions. In times of crisis, in turn, it is women -and dissidences- who assume most of the responsibilities in maintaining life, doing everything to guarantee subsistence. When it comes to academic life, some studies show, for example, that in Brazil (Alves et al., 2022) while white men have raised the rate of their production, women, particularly mothers, have been the most affected. They have reduced or paralyzed their production or have requested scholarships and subsidies while dealing with various situations derived from the cis-heteropatriarchy itself.

The inequalities in the academic and scientific field are reflected not only in the number of publications that emerge from research teams based in countries of the global north but also in an academic “extractivism” according to which we, members of countries of the global south, provide the “raw material.” From decolonial feminism, this has been widely questioned. However, we are constantly witnessing extractivist logic and we observe little or no reflection on it in the central countries. There is a triple condition of devaluation: being from the Global South, being from the social sciences, and being women (and dissidents). This triple devaluation, for its part, almost directly implies triple extractivism. As “peripheral” countries: we export our reflections, the raw material with which the central countries boast of analyzing using their categories; an extractivism of reflections that come from the field of social sciences committed and involved with the populations and in contact with the territory and, finally, the exploitation of the female labor force. The field of qualitative research was not exempt from these issues.

## Conclusion: Opportunities and challenges doing research during the pandemic

Doing research during the COVID-19 pandemic undoubtedly was an enormous challenge for all of us, especially in the case of women who must take care of children or elders.

Despite that fact, in southern countries, as we have shown in the Mexican, Brazilian, Chilean, and Argentinian cases the difficulties are before the pandemic outbreak. The scarcity of resources, the precarious funds obtained to hold the research team and the peripheric position in knowledge production conditions was the starting point for doing Rapid Qualitative Research.

In that sense, with the reflexive exercise of making ourselves some questions about the urgency, we do not lose sight of other priorities, which demand increasingly urgent attention.

- What will be done? Why? For whom?
- How to do this research? Which are the conditions of social knowledge production?
- Who is doing/will be doing the research in those conditions?

None of those questions are new or specific to pandemic times, all of them are problems that are daily breakthroughs for all of us as academics or researchers in Latin America, even though the pandemic context within its urge and conjunction required a rapid response and made more visible the situation and dilemmas we outlined here.

Even so, the meeting of all of us was possible due to the RREAL invitation to do this collaborative research during the pandemic outbreak, also this publication was possible due to the RREAL financial aid, and it was in this context that our meeting and reflections were possible. This participation is also evaluated positively in our academic contexts.

At a local level, we can highlight some points that help us to develop present research and that would be useful for future research: In the first place, the urgency of strengthening the ethical committee to fasten the project evaluation, as well as to take extraordinary policy measures to help us to do ethical research in extraordinary contexts like the pandemic one.

Second, to develop special financing opportunities for social sciences, that include gender-equal conditions. We note that a good way to keep a research team is to work with postgraduate students doing their thesis in the frame of the major research, which also represents a growing space for learning.

Third, to promote publications in the languages of researchers' origin means to have the opportunity for writing in our mother tongue and translating to other languages, and to do the same with English speakers' researchers to publish in two or three languages. That is, to promote easier access to information by local researchers, general populations, and, especially, policymakers. It also promotes the development of shared research between north and south researchers. This collaboration should involve data collection analyzed by researchers from the country of origin, autonomy for publication, and language diversity, as the RREAL project network.

## Data availability statement

The original contributions presented in the study are included in the article/supplementary material, further inquiries can be directed to the corresponding author/s.

## Author contributions

All authors listed have made a substantial, direct, and intellectual contribution to the work and approved it for publication.

## Funding

RREAL funded the publication of the article. The Argentinean team was funded by the National Agency of Promotion of Research, Technical Development and Innovation (Agencia I+D+i). The Brazilian interviews were carried out within the framework of ongoing postdoctoral ethnographic research (FAPESP Process: 2019/13439-7). The Chilean team received a grant from the National Agency of Research and Development (ANID) (Proyecto ANID COVID 0383).

## Conflict of interest

The authors declare that the research was conducted in the absence of any commercial or financial relationships that could be construed as a potential conflict of interest.

## Publisher's note

All claims expressed in this article are solely those of the authors and do not necessarily represent those of their affiliated organizations, or those of the publisher, the editors and the reviewers. Any product that may be evaluated in this article, or claim that may be made by its manufacturer, is not guaranteed or endorsed by the publisher.

## Footnotes

^1^Ministerio de Salud de la Nación. Resolución 908/2020. Pautas Éticas y Operativas para la Evaluación Ética Acelerada de Investigaciones en Seres Humanos relacionadas con COVID-19 Disponible en: http://servicios.infoleg.gob.ar/infolegInternet/anexos/335000-39999/337359/norma.htm.

^2^For more information: https://www.argentina.gob.ar/ciencia/agencia/acciones-covid-19/pisac-covid-19/pisac-covid-19-no77-los-nuevos-escenarios-en-la?tca=KPII_a_UEMk2Ou7AURNqd1JY-JRnUKeleo_4iOKBZcI.

^3^For more information (see Bautista-González et al., 2021).

^4^For more information (see ANID, 2020).

^5^ANID (2020). *Ministerio de Ciencia y ANID dan a Conocer Seleccionados del Fondo de Investigación Cient*í*fica COVID-19*. Retrieved from: https://www.anid.cl/blog/2020/06/01/ministerio-de-ciencia-y-anid-dan-a-conocer-seleccionados-del-fondo-de-investigacion-cientifica-covid-19/.
